# Evaluation of collector spacing, positioning, and nozzle selection methods on water distribution uniformity in center pivots

**DOI:** 10.1371/journal.pone.0331122

**Published:** 2025-09-12

**Authors:** Lucas Maltoni Andrade, Rubens Alves de Oliveira, Fernando França da Cunha, Luis César Dias Drumond, Paulo Roberto Cecon, Job Teixeira de Oliveira, Catariny Cabral Aleman

**Affiliations:** 1 Department of Agricultural Engineering, Center of Agricultural Sciences, Federal University of Viçosa, Viçosa, Minas Gerais, Brazil; 2 Institute of Agricultural Sciences, Federal University of Viçosa, Rio Paranaíba Campus, Minas Gerais, Brazil; 3 Department of Statistics, Center of Exact Sciences, Federal University of Viçosa, Viçosa, Minas Gerais, Brazil; 4 Department of Agronomy, Federal University of Mato Grosso do Sul, Chapadão do Sul Campus, Mato Grosso do Sul, Brazil; Shahrekord University, IRAN, ISLAMIC REPUBLIC OF

## Abstract

Proper design and periodic evaluation of center pivot irrigation systems are essential for ensuring uniform water distribution and improving water use efficiency in irrigated agriculture. This study assessed the impact of collector spacing and sampling methods, including a single-level collector line and combinations of multiple lines at different irrigated locations, on water distribution uniformity. Additionally, strategies for selecting emitter nozzles were analyzed. Uniformity assessments were conducted on two center pivots (P180 and P360) using collectors spaced at 1 m, with simulated spacings of up to 12 m by omitting intermediate readings. Collector lines were placed perpendicular to the pivot’s movement at 50% regulation and were evaluated three times. Data were analyzed individually for each line position and in groups, following layout recommendations from the literature. The mean water depth (L_M_), weighted mean depth (L_W_), and modified Christiansen’s uniformity coefficient (CUC_HH_) were used as evaluation metrics. Flow rate measurements were performed, with three repetitions per nozzle number. Nozzle selection considered the required flow rate and nozzle diameter for both constant and doubled spacing between emitters in the first two spans. Measured flow rates resulted in higher simulated water depths and lower CUC_HH_ values compared to simulated reference flow rates. However, the average field CUC_HH_ exceeded 90% for both pivots, being classified as excellent. Evaluation methods combining different collector line positions showed no significant differences between means, nor did the combined effect of collector spacing and single-line positioning. No significant differences were found between collector spacings alone. However, collector line positioning influenced results, with P180 showing differences in L_W_ and P360 in L_M_, L_W_, and CUC_HH_. Estimated evaporation and wind drift losses were 14.21% for P360 and 13.49% for P180. The evaluated nozzle selection combinations showed a theoretical CUC_HH_ higher than the simulated values based on the original nozzle listing for both pivots.

## Introduction

The effectiveness of water distribution in irrigation is an increasingly relevant topic, as its efficient management is essential for agricultural productivity and rational water use. In 2022, according to [1], irrigated agriculture accounted for 50.4% of all water withdrawals in Brazil, equivalent to approximately 1,027 m³s ⁻ ¹. Various irrigation systems contribute to this consumption, with center-pivot irrigation deserving special attention due to its rapid expansion [2].

Center pivots consist of a lateral pipeline supported by mobile towers that rotate around a central axis, irrigating a circular area [3]. These systems are known for their high uniformity potential due to significant water overlap between emitters and the slow movement of the lateral line [4]. However, as noted by [5], many irrigation systems often operate below their potential efficiency, largely due to poor management. Also, field water distribution is not perfectly uniform, as it can be affected by environmental factors such as wind drift and evaporation. It may also be influenced by design flaws, or operational and maintenance deficiencies, potentially leading to areas receiving either excessive or insufficient amounts of water [6]. These irregularities can reduce profitability and place greater pressure on already limited water resources [7,8]. In light of this, assessing irrigation system performance through field evaluations conducted before and during the growing season is essential not only to improve operational efficiency, but also to enhance system design and support informed decision-making [4,5,9].

In order to identify water depth variations along the lateral line, uniformity evaluations should follow the standard procedures recommended in the literature [10,11]. These involve placing at least two radial lines of identical water collectors, positioned at a constant height and spaced 3–5 meters apart. Additionally, the distance between the ends of the radial collector lines should not exceed 50 meters. Previous versions of [10] also indicated an angular spacing of 3 degrees between the lines, as long as the final distance between their ends did not surpass the 50-meter limit. However, despite these established guidelines, alternative methods and adaptations for assessing water distribution uniformity are also found in the literature. Protocols and results often vary due to diverse field conditions and pivot configurations. Several reported approaches include: the positioning of collector lines along both the steepest uphill and downhill directions in order to capture the potential influence of pressure variations along the lateral line, intensified by the terrain slope [12]; the use of four collector lines arranged perpendicular to each other, providing data from multiple pivot radii for a more representative uniformity assessment [2]; alternative collector arrangements, such as meshed and circular layouts, to account for wind drift, stoppage effects and varying topography [13]; distinct collector sizes and installation heights [14]; and variable spacing, with the distance between collectors decreasing towards the end of the line [15].

In practice, however, evaluations are frequently conducted using a single line of collectors along one radius [16], since more detailed setups encouraged by both technical guidelines and academic literature typically require greater allocation of personnel and material resources, as well as time investment. This effort becomes even more significant when multiple pivots must be evaluated. Moreover, collector spacing can be challenging in larger pivots, where maintaining recommended intervals further increases these demands. Consequently, practitioners also often use wider collector spacing to mitigate such issues. This common gap between theoretical recommendations and practical constraints highlights the need for evaluation strategies that are both applicable and efficient.

Considering this context, the present study compares a selection of collector line positioning configurations, as described in existing guidelines and prior studies, alongside variations in container spacing, to evaluate their combined influence on uniformity assessment results. Although some studies have addressed these variables separately or in more limited contexts, comprehensive evaluations exploring their combined effects remain scarce. To fill this gap, extensive tests were conducted on two center pivots across multiple radii, encompassing varying terrain characteristics, and featuring collector lines with containers spaced at 1 m intervals. Subsets of the resulting data were then analyzed to simulate wider spacing scenarios, enabling direct comparison of various configurations.

In addition to assessing the performance of installed systems through field evaluations, it is equally important to address decisions made during the design phase. Among these, emitter and nozzle diameter selection, commonly referred to as the nozzle package, plays a key role in achieving suitable water distribution. According to [17], various commercial options can deliver good results. However, [18] emphasizes that proper selection is crucial for maximum efficiency. Factors such as the desired water depth, terrain profile, and crop growth stages, among others, must be considered when selecting the most optimal nozzle package [14] in order to maintain high water distribution uniformity. This, in turn, can impact crop productivity and influence operational costs, as previously stated.

Despite the importance of proper nozzle selection, the literature lacks studies that explore selection criteria and their implications. Manufacturers generally provide predefined nozzle packages [19], yet the underlying principles are not commonly disclosed, highlighting the need for further investigation. Therefore, this study also aims to evaluate the effects of nozzle selection methods on theoretical water distribution uniformity values.

## Materials and methods

**Fig 1:**
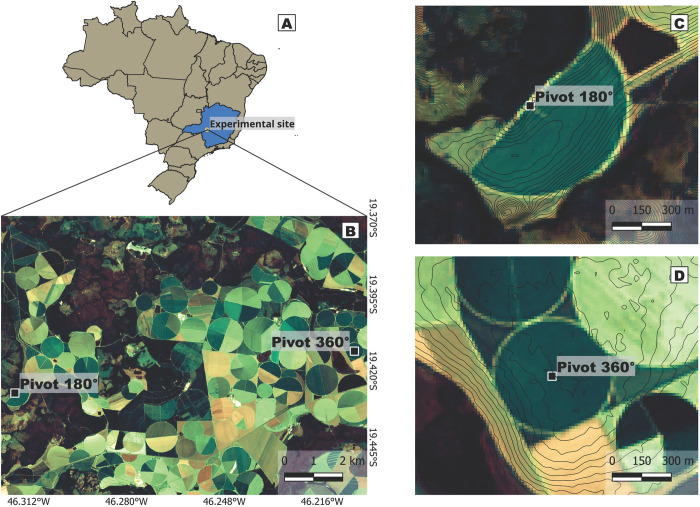
Location of the central pivots. Experimental site in relation to Minas Gerais, Brazil (A), overview of the study area (B), focused visualization of the analyzed center pivots (C and D). Contour lines derived from the Copernicus GLO-30 digital elevation model. Basemap: Fused Landsat 9 image combining the panchromatic and visible bands. Source: USGS – The figure was elaborated using QGIS [21].

The trials were conducted in Rio Paranaíba, Minas Gerais, Brazil, at Aliança Farm (collaborating organization), located at 19°24’10’‘S and 46°15’41’‘W, as shown in Fig 1, from mid-September to mid-October 2023. Altimetric profiles for terrain characterization were obtained using digital elevation models from the European Copernicus program, with a spatial resolution of 30 × 30 m. According to the Köppen classification, the region’s climate is Cwb, characterized by temperate summers and dry winters [20].

Two center pivots were analyzed (Table 1), namely the pivot 180° (P180) and the pivot 360° (P360), both with brachiaria in an early growth stage. It was reported that these systems have been in operation for over 25 years. However, determining their actual operational age was challenging, as numerous preventive and corrective maintenance interventions such as part replacements and even disassembly and relocation to different areas have been carried out over time.

**Table 1:** Specifications of the pivots (P180 and P360).

Characteristics	P180	P360
Composition	10 spans and an overhang	6 spans and an overhang
Area (ha)	35.3	28.5
Flow rate (m³ h ⁻ ¹)	206.58	186.99
Revolution time_100% speed_ (hours)	10.25	6.68
Projected water depth_100% speed_ (mm)	2.71	4.39
Emitters	193	111
Nozzle diameter range (mm)	2.38 - 8.94	2.38 - 10.32
Final exit	Final spray (Senninger)	R55i VT, N° 80 (Nelson)

Technical specifications provided by the collaborating organization.

**Fig 2:**
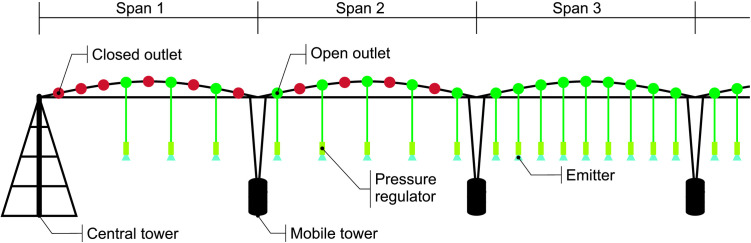
Emitter configuration in the initial spans of pivots P180 and P360.

The center pivots were equipped with I-Wobler emitters, installed approximately 1.83 m above ground, with a 2.29 m spacing. Pressure-regulating valves were set to 7 mWC (meters of water column). The first three outlets on the initial span of P180 and P360 were closed due to their proximity to the central tower. Additionally, the last nozzle at the overhang of both pivots had a disproportionately larger diameter than the preceding one. For the first two spans of each pivot, emitter outlets were alternated, reducing water overlap, as shown in Fig 2.

**Fig 3:**
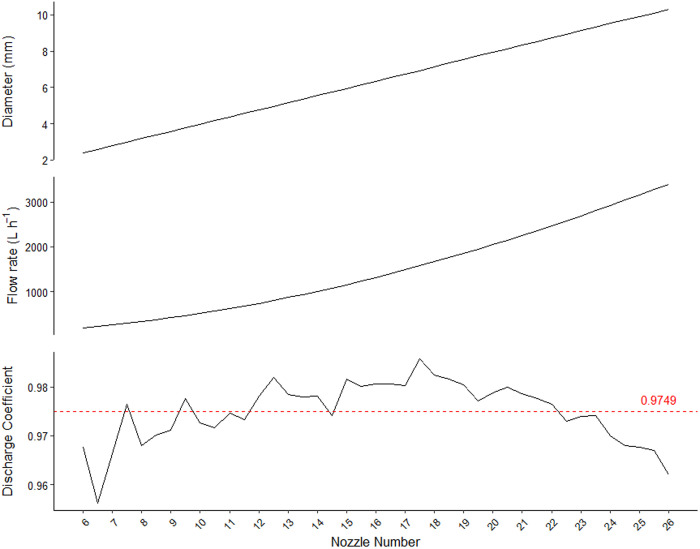
Diameter, flow rate, and discharge coefficient by nozzle number.


The nozzle diameters and expected flow rate values (Q_reference_) are shown in Fig 3. The discharge coefficient (Cd) was also included, calculated using Equation (1):

$$\begin{array}{c} {Cd}_{i}\;=\;\frac{4\;q_{i}}{\pi {{\;D}_{i}}^{2}\;\sqrt{2\;g\;P_{S}}} \end{array}$$ (1)

Where Cd_i_ is the discharge coefficient of nozzle ‘i’, which is dimensionless, D_i_ is the diameter of nozzle ‘i’ (m), q_i_ is the flow rate of nozzle ‘i’ (m³ s ⁻ ¹), g is the acceleration due to gravity (m s ⁻ ²) and P_s_ is the service pressure (7 mWC).

### Field assessments

The flow rate of one emitter for each nozzle size was measured (Q_measured_). A total of 29 emitters from P180 and 33 from P360 were evaluated, each with three repetitions. During the measurement process, transparent 20-liter buckets, a timer with a precision of one-tenth of a second, a graduated 1-liter measuring cylinder, and a closed container to direct all emitted water into the bucket were used. The flow rate was determined by dividing the measured volume by the filling time. Q_measured_ was then compared to Q_reference_, previously presented in Fig 3.

To measure the water depth applied by the center pivots, identical water collectors were used, each 10.2 cm in height with a collection area of 56.2 cm². They were installed approximately 0.7 m above ground level and positioned perpendicular to the movement of the pivot. Readings were taken using a transparent graduated cylinder with a 1.0 mL scale. Water depth was calculated by dividing the collected volume by the collector area.

Four collector line configurations were tested: a single leveled line (MA_single_), often employed in practical field evaluations; two lines angled at 3° relative to each other (MA_ABNT_), based on recognized standard guidelines [10,11]; three lines, with the first positioned along the steepest uphill direction, the second along the steepest downhill direction, and the third on the leveled ground (MA_3 lines_); and four lines perpendicular to each other (MA_4 lines_), both latter setups reported in the literature [2,12]. In addition, these multi-line configurations allow for broader spatial coverage and more representative sampling across the irrigated area, making them particularly suitable for comparative analysis.

Due to the semicircular nature of P180, only the MA_single_, MA_ABNT_, and MA_3 lines_ evaluation methods were applied. In the MA_3 lines_ configuration, the two lines positioned in irregular regions were subjected to both uphill and downhill terrain.

For P360, MA_3 lines_ could not be fully implemented since no uphill area within the pivot occurred. Instead, only two collector lines, L2 and L4, were used. As a result, the modified method for P360 was designated as MA_3 lines_*.

**Fig 5:**
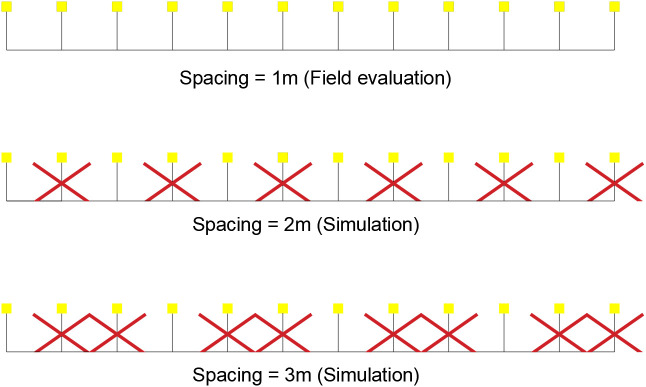
Data exclusion process for collector spacing simulation.

Collectors were installed at 1 m spacing for each position. After field data collection, uniformity values were determined for the 1 m spacing, and larger spacings were simulated by omitting readings from collectors placed at intervals shorter than the simulated distance (Fig 5).

Integer spacing values were simulated up to a maximum distance of 12 m. Three repetitions were performed for each of the previously mentioned collector lines, with each pivot completing three full rotations, totaling 12 tests for P180 and 15 tests for P360.

During testing, collected water depths were based on a pivot speed set to 50% of the percent time. According to [10,11], pivot speed should be adjusted to apply a water depth of at least 15 mm unless otherwise specified by the client or partner. Given the number of planned tests, a 50% regulation was considered the most appropriate. This approach prevented excessive water application and ensured that the full rotation of the equipment could be completed within 6 to 18 hours on the same day if necessary.

The distribution uniformity coefficient, mean water depth (L_M_), and weighted mean water depth (L_W_) were calculated for each trial. For uniformity evaluation methods involving multiple collector lines (MA_ABNT_, MA_3 lines_, and MA_4 lines_), uniformity and water depth values were first computed individually for each line. The average values across the collector lines within each method were then determined.

Once the original nozzle list for the pivots, provided by the collaborating organization, was validated in the field, theoretical uniformity was also estimated using water depths based on Q_reference_ and Q_measured_.

The method proposed by [22] and modified by [23], Equation (2), was adopted to calculate water distribution uniformity in center pivots:


$$\begin{array}{c} {CUC}_{HH}\;=\left(\;1\;-\;\frac{\left(\sum_{j=1}^{N}R_{j}\left|\;X_{j}\;-\;{\overset{―}{X}}_{p}\right|\right)}{\sum_{j=1}^{N}X_{j}\;R_{j}}\right)\;100 \end{array}$$ (2)

Where X_p_ is given by:


$$\begin{array}{c} {\overset{―}{X}}_{p}\;=\;\frac{\sum_{j=1}^{N}X_{j}\;R_{j}}{\sum_{j=1}^{N}\;R_{j}} \end{array}$$ (3)

Where CUC_HH_ stands for Christiansen’s Uniformity Coefficient [22], modified by Heermann and Hein [23], and expressed as a percentage (%), N is the number of observations, X_j_ is the water depth applied at point j (mm), X_p_ is the weighted mean water depth (mm), and R_j_ is the distance from collector j to the center of the pivot (m).

CUC_HH_ was also used for qualitative classification to assess water distribution uniformity, following the criteria outlined in Table 3.

**Table 3:** Classification of Christiansen´s Uniformity Coefficient (CUC).

Classification	CUC (%)
Excellent	> 90%
Good	80–90%
Regular/Poor	80–60%
Unacceptable	< 60%

Adapted from [24] and [25].

Before each trial, wind speed, temperature, and relative humidity were repeatedly measured at a height of 2 m using a portable digital anemometer (model PM6252A). Trials were postponed when initial wind speed consistently exceeded 3.5 m s ⁻ ¹, remaining on hold until more favorable conditions were met.

Due to the 1-meter spacing between collectors and the considerable length of the pivots, reading time could not be overlooked. Recognizing its importance, as highlighted by [14], a correction was applied to minimize measurement errors caused by water evaporation within the collectors. Three control collectors were placed near the pivots, each filled with 100 mL of water at the start of the trial. Once readings were completed, the final volume was measured, and total evaporation depth was determined by the difference between initial and final volumes.

Reading times were periodically recorded to estimate each collector’s exposure to evaporation after the pivot passed. This enabled applying an evaporation correction factor per collector, Equation (4), assuming an average reading time of 10 seconds each:


$$\begin{array}{c} {Volume}_{corr}=\;Volume\;+\;\frac{\overset{―}{\Delta Evap}}{{Time}_{test}}*{Time}_{exposure} \end{array}$$ (4)

Where Volume_corr_ is the corrected water volume (mL), Volume is the measured water volume (mL), $\overset{―}{∆\text{Evap}}$ is the difference between the initial and final volumes of the evaporation control collectors (mL), Time_test_ is the duration for which control collectors remained exposed (seconds), and Time_exposure_ is the duration for which collectors within the pivot’s reach remained exposed after its passage (seconds).

Notably, for the two collector lines angled 3° apart, the second irrigated line could only be read after completing measurements for the first. This delay resulted in longer exposure times for the second line.

Water losses during the application process were estimated based on the average L_W_ at each evaluated position, using the projected depths at a 50% percent timer setting as reference (Table 4).

**Table 4:** Projected total water depths for different percent timer settings.

	Projected total water depth (mm)		
	100%	75%	50%
**P180**	2.71	3.61	5.41
**P360**	4.39	5.85	8.78

Technical specifications provided by the collaborating organization.

Evaporation and wind drift losses (DE_L_) were estimated using the following equation:


$${DE}_{L}=\left(1-\left(\frac{L_{W}}{L_{P}}\right)\right)*100$$ (5)

Where DE_L_ represents evaporation and wind drift losses (%), L_W_ is the weighted mean water depth collected (mm), and L_P_ is the projected total water depth (mm).

### Nozzle selection

**Fig 6:**
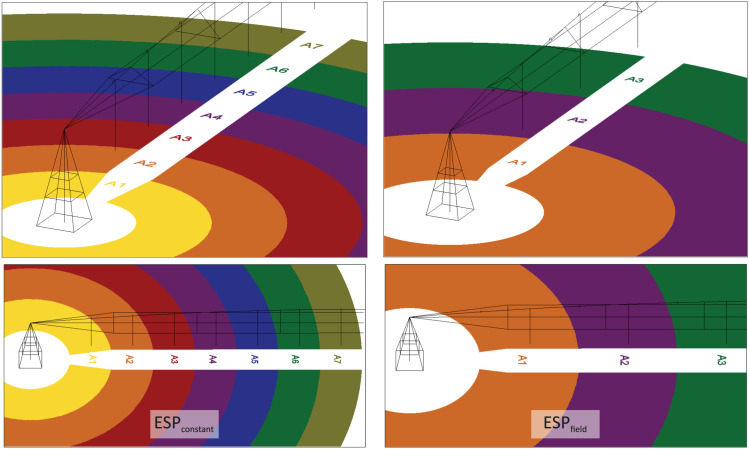
Annular emitter areas for constant spacing (ESP_constant_) and doubled spacing in the first two spans (ESP_field_).

Two spacing conditions were analyzed: one with uniform emitter spacing along the lateral line (ESP_constant_) and another with doubled spacing in the first two spans (ESP_field_) (Fig 6). The selection methods, based on the approximate flow rate (MQ_ap_) and the average discharge coefficient (MCd), aimed to determine the theoretical CUC_HH_ through an approximate reproduction of the pivot’s design specifications. This process aimed to achieve the projected total water depth for a full rotation with the percent timer set at 100% for both pivots (Table 4).

The process initially involved calculating the water volume required for each annular area using Equation (6):


$${V_{water}}_{i}={LP}_{100\%}*{{Area}_{annular}}_{i}$$ (6)

Where ${{\text{V}}_{\text{water}}}_{\text{i}}$ is the volume of water to be applied by emitter ‘i’ (L), LP_100%_ is the projected total water depth equivalent to the percent timer regulated at 100% (mm), and ${{\text{Area}}_{\text{annular}}}_{\text{i}}$ is the area to be irrigated by emitter ‘i’ (m²).

Next, the required flow rate (Q_required_) for each pivot outlet was calculated using Equation (7), considering the time needed for a full rotation with the percent timer set to 100%. Finally, for MQ_ap_, nozzles were selected based on the reference flow rate closest to Q_required_.


$$\begin{array}{c} {Q_{required}}_{i}\;=\;\frac{{V_{water}}_{i}}{T_{100\%}} \end{array}$$ (7)

Where ${{\text{Q}}_{\text{required}}}_{\text{i}}$ is the flow rate required by emitter ‘i’ (L h^−1^), ${{\text{V}}_{\text{water}}}_{\text{i}}$ is the volume of water to be applied by emitter 'i' (L), and T_100%_ is the time for a full rotation of the pivot with the percent timer set to 100%.

For MCd, the average of the Cd_i_ values (Fig 3) was used to calculate the required nozzle diameter at each position along the lateral line, following Equation (8). This approach was necessary because an individual discharge coefficient could not be assigned initially due to the absence of a predefined nozzle. Finally, the nozzle with the closest available diameter to the calculated value was selected:


$$\begin{array}{c} D_{i}\;=\;\sqrt{\frac{4\;q_{i}}{\pi \;\sqrt{2\;g\;P_{S}}{\;Cd}_{mean}}} \end{array}$$ (8)

Where D_i_ is the diameter of the nozzle ‘i’ (m), q_i_ is the flow rate of nozzle ‘i’ (m³ s ⁻ ¹), g is the acceleration due to gravity (m s ⁻ ²), P_s_ is the operating pressure (7 mWC), and ${\text{Cd}}_{\text{mean}}$ is the average of the Cd_i_ values (dimensionless).

The MQ_ap_ and MCd analysis involved simulating the installation of the selected nozzles for P180 and P360 and their projected water depths based on Q_reference_. This approach enabled a comparison between the theoretical uniformity results obtained from these methods, the uniformity assessed in field tests, and the theoretical uniformity based on Q_measured_ and Q_reference_ according to the original nozzle list. Also, all simulations were conducted exclusively for the nozzles, not considering the final spray for P180 and R55i for P360 (Table 4), as they were post-design additions.

### Statistical analysis

The experimental analysis was conducted separately for each pivot, using a 5 × 12 factorial scheme for P360 and a 4 × 12 factorial scheme for P180, both in a randomized block design (RBD) with three repetitions. Blocks were incorporated to account for variation between repetitions, helping to mitigate the effects of climatic fluctuations and other possible unforeseen interferences.

Two factors were analyzed: (i) the position of the collector lines in the field, with five levels for P360 and four for P180, and (ii) the spacing between collectors, with 12 levels for both pivots. Additionally, a separate analysis was performed to compare different methods for evaluating water distribution uniformity. This analysis followed an RBD framework, where the sole factor represented the various methods derived from the combinations of collector line positions, as shown in Table 2.

**Table 2:** Combinations of individual collector line positions (L1 – L5).

	Methodologies	
	P180	P360
**Positions**	L1 + L2 + L3 (MA_3 lines_)	L1 + L2 + L3 + L4 (MA_4 lines_)
		L2 + L4 (MA_3 lines_*)
	L3 + L4 (MA_ABNT_)	L4 + L5 (MA_ABNT_)
	L3 (MA_single_)	L4 (MA_single_)

MA_single_: a single leveled line; MA_ABNT_: two lines angled at 3° relative to each other; MA_3 lines_: three lines, with two subjected to both uphill and downhill terrain, and one on leveled ground; MA_3 lines_*: modified version applied to P360 using only two lines due to the absence of an uphill area; MA_4 lines_: four lines arranged perpendicularly.

Data were analyzed using analysis of variance (ANOVA), and significant differences were compared using Tukey’s test at a 5% significance level. Statistical analyses were performed in R [26] using the ExpDes.pt package [27] for ANOVA and multiple mean comparison tests.

## Results and discussion

### Measured and reference flow rates

**Fig 7:**
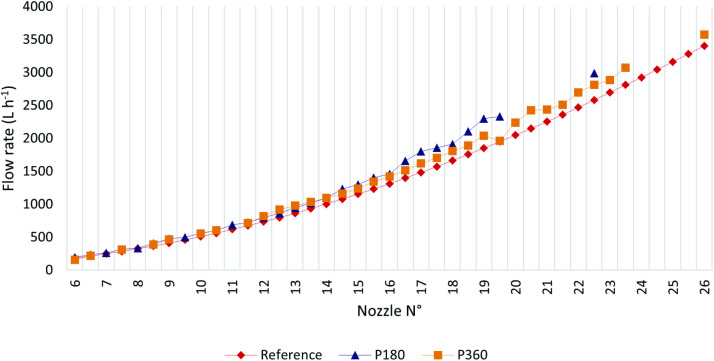
Measured flow rates (Q_measured_) for P180 and P360 (blue and orange lines) compared to reference values (Q_reference_) at 7 mWC (red line).

Fig 7 presents Q_measured_ for both pivots alongside Q_reference_ for each nozzle number. Columns without Q_measured_ values for either pivot indicate that the corresponding nozzle number was not used in the pivot with missing data. Notably, most Q_measured_ values for P180 and P360 exceed Q_reference_ at a pressure of 7 mWC.

**Fig 8:**
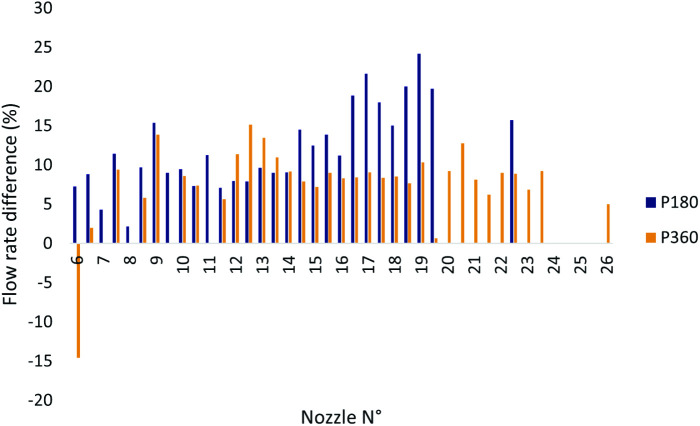
Percentage difference between Q_measured_ and Q_reference_ (baseline) flow rates for P180 and P360.

It was reported that the pressure-regulating valves in both pivots had been operating for a long time and were due for replacement, likely causing this issue. Q_measured_ values for P180 nozzles were not only higher than Q_reference_ but also exceeded Q_measured_ values for P360 from approximately nozzle position 14.5 onward. Fig 8 shows the percentage differences between Q_measured_ for P180 and P360, using Q_reference_ as the baseline.

Nozzles No. 6, 6.5, and 19.5 for P360 and No. 8 for P180 exhibited irregular behavior, particularly No. 6 and 19.5. Nozzle 6 showed a significant drop, with Q_measured_ approximately 14.5% lower than Q_reference_, while No. 19.5 had a value nearly equal to Q_reference_. This may be attributed to partial nozzle clogging that was not detected during field measurements.

**Fig 9:**
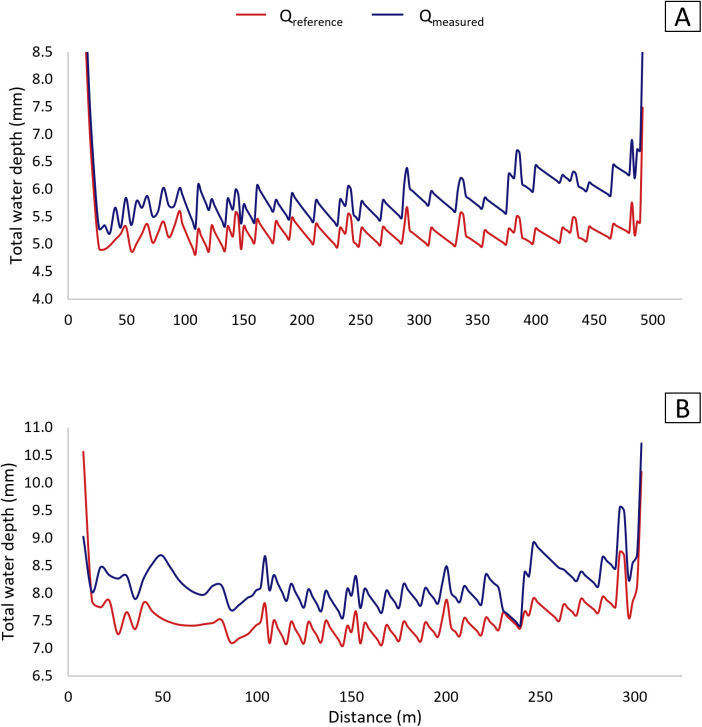
Total water depths for P180 (A) and P360 (B) based on reference (Q_reference_) and measured (Q_measured_) flow rates.

Fig 9 presents the simulated total water depth pattern along the radii of P180 and P360 based on Q_measured_ and Q_reference_ for the percent timer set at 50%. Q_measured_ values were assumed to be equal for nozzles of the same number across different emitters.

**Fig 4:**
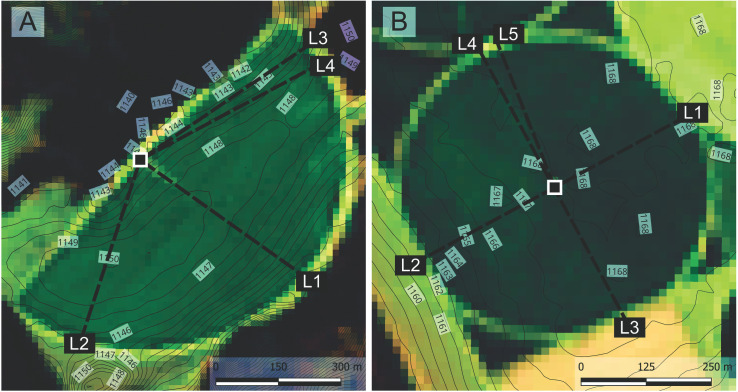
Collector line positions for P180 (A) and P360 (B). Contour lines derived from the Copernicus GLO-30 digital elevation model. Basemap: Fused Landsat 9 image combining the panchromatic and visible bands. Source: USGS – The figure was elaborated using QGIS [21].

For Q_measured_ in P180 (Fig 9A), a systematic increase in simulated depths is observed from approximately 300 meters onward. This trend aligns with Figs 7 and 8, where nozzle 14.5 and subsequent nozzles show higher flow rates, corresponding to their installation distance. At the time of measurement, P180 was positioned near L1 (Fig 4A), where the lateral line experiences a steep drop in elevation beyond 300 meters. This elevation alteration likely contributed to higher flow rates and, consequently, greater water depths.

Total water depths for both pivots exceeded those based on Q_reference_ values, as the irrigated area per emitter remained constant (Table 5). Additionally, depths were notably higher at the start and end of the lateral line compared to the average. At the beginning of the pivot, initial nozzle diameters were larger than necessary, as the required sizes were too small to be met, while at the end, the last nozzle had a disproportionately larger diameter than the preceding one, further increasing application depth.

**Table 5:** Weighted total water depth (L_W_) and modified Christiansen’s uniformity coefficient (CUC_HH_) for measured (Q_measured_) and reference (Q_reference_) flow rates.

	P360		P180	
	L_W_ (mm)	CUC_HH_ (%)	L_W_ (mm)	CUC_HH_ (%)
**Q**_**reference**_	7.59	96.25	5.22	97.12
**Q**_**measured**_	8.22	95.69	5.96	95.00

L_W_ values based on Q_measured_ increased by 8.30% for P360 and 14.17% for P180 compared to Q_reference_. In contrast, CUC_HH_ decreased by 0.58% for P360 and 2.18% for P180. However, it is important to emphasize that the simulated water depths for Q_measured_ and Q_reference_ were lower than the projected total water depth at 50% setting (Table 4) for P360 (−0.56 and −1.19 mm), whereas for P180, the variations were +0.55 and −0.19 mm, respectively. Such differences may be due to nozzle packages that do not match the intended water depth, as well as a result of the approximate reproduction of the pivots’ design specifications for this simulation, which may lead to discrepancies.

An atypical variation in total application depths was observed for P360 (Fig 9B) between 50 and 100 meters from the pivot point. This irregularity occurred for Q_measured_ and Q_reference_, as the application depth did not follow the expected pattern. The anomaly is likely due to successive rounding to larger flow rates during the original nozzle selection within this interval.

**Fig 10:**
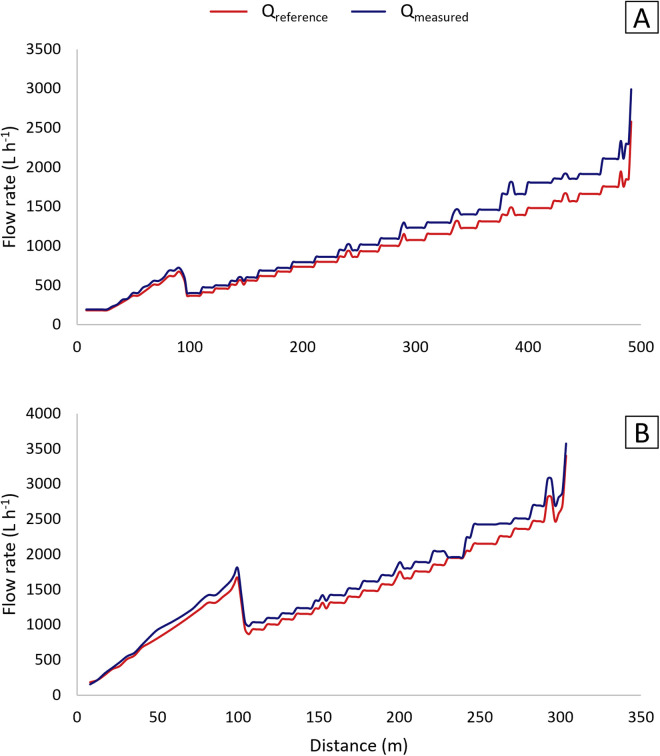
Measured (Q_measured_) and reference (Q_reference_) nozzle flow rates along the lateral lines of P180 (A) and P360 (B).

The flow rate progression for both pivots followed the expected pattern (Fig 10), with flow rates increasing as emitter nozzle diameters increase farther from the center [19]. In some pipeline sections, emitters with the same nozzle diameter were used for progressively larger areas due to the unavailability of commercial diameters matching the required values. This same factor explains the fluctuating pattern observed in Fig 9, where total application depth varies as a result of a fixed flow rate to a continuously changing area.

In Fig 10, an abrupt reduction in flow rate near 100 m is observed in both cases. This drop resulted from a change in emitter spacing, as shown in Fig 2 and also noted by [19], in order to maintain the desired water depth.

### Selected nozzles

**Fig 11:**
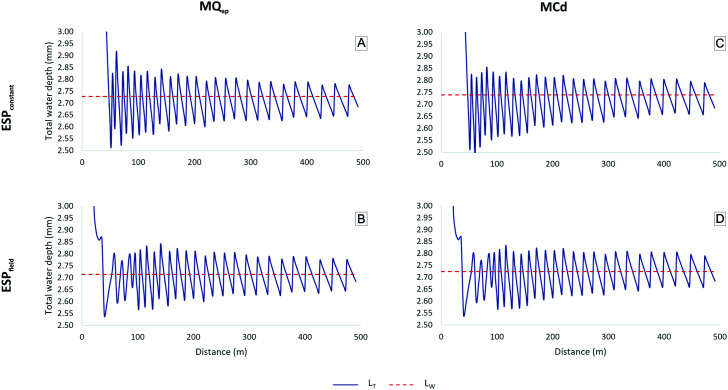
Weighted (L_W_) and total water depths (L_T_) of P180 for ESP_constant_ and ESP_field_ based on nozzle selection criteria MQ_ap_ and MCd. ESP_constant_: uniform emitter spacing along the lateral line; ESP_field_: doubled emitter spacing in the first two spans; MQ_ap_: nozzle selection method based on the approximate flow rate; MCd: nozzle selection method based on the average discharge coefficient.

**Fig 12:**
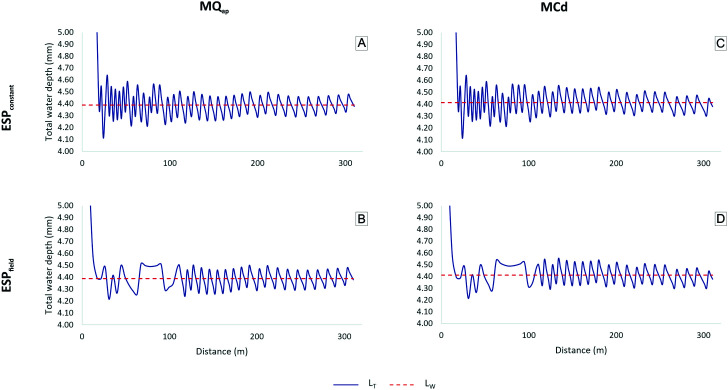
Weighted (L_W_) and total water depths (L_T_) of P360 for ESP_constant_ and ESP_field_ based on nozzle selection criteria MQ_ap_ and MCd. ESP_constant_: uniform emitter spacing along the lateral line; ESP_field_: doubled emitter spacing in the first two spans; MQ_ap_: nozzle selection method based on the approximate flow rate; MCd: nozzle selection method based on the average discharge coefficient.

Figs 11 and 12 show variations in water depth at 100% percent timer regulation along the radius of P180 and P360 for the nozzle selection methods MQ_ap_ and MCd. Additionally, both methods were applied to ESP_constant_ and ESP_field_, resulting in four distinct arrangements.

The total depths applied by emitters closer to the pivot point showed greater variation from L_W_, as previously discussed in Fig 9. As Q_reference_ approaches Q_required_, the depths gradually converge to the average value.

Figs 12B and 12D reveal an atypical variation in depth between 50 and 100 m for both MQ_ap_ and MCd under the ESP_field_ condition. This variation was not observed under ESP_constant_ (Figs 12A and 12C). However, a similar distortion was previously identified for P360 (Fig 9B) within the same distance range, based on the nozzle list provided by the collaborating organization. This suggests that, as previously discussed, rounding up to larger reference flow rates also occurred in the MQ_ap_ and MCd nozzle selection methods.

Table 6 presents L_W_ and CUC_HH_ values simulated for each approach using Q_reference_. All methods produced total depths closely matching the project specifications (Table 4). CUC_HH_ values were higher for all methods compared to those obtained in simulations based on the original nozzle list provided by the collaborating organization for both Q_reference_ and Q_measured_ (Table 5). Although these results stem from a simulation, [6] reported significant uniformity improvements in field evaluations after replacing sprinkler packages, underscoring the importance of retrofitting.

**Table 6:** Weighted total water depth (L_W_) and modified Christiansen’s uniformity coefficient (CUC_HH_) for nozzle selection simulations of P180 and P360 emitters.

		MQ_ap_		MCd	
		L_W_ (mm)	CUC_HH_ (%)	L_W_ (mm)	CUC_HH_ (%)
**ESP**_**constant**_	**P180**	2.73	97.58	2.74	97.58
**ESP**_**field**_		2.71	98.27	2.72	98.26
**ESP**_**constant**_	**P360**	4.39	98.49	4.41	98.47
**ESP**_**field**_		4.39	98.70	4.41	98.69

ESP_constant_: uniform emitter spacing along the lateral line; ESP_field_: doubled emitter spacing in the first two spans; MQ_ap_: nozzle selection method based on the approximate flow rate; MCd: nozzle selection method based on the average discharge coefficient.

The different combinations yielded similar total water depth and CUC_HH_ values. However, both MQ_ap_ and MCd under the ESP_field_ condition resulted in a slightly higher, though likely negligible, theoretical CUC_HH_ compared to ESP_constant_. Additionally, in the ESP_field_ scenario, the need for components such as emitters and pressure-regulating valves is reduced. While ESP_field_ lowers acquisition and operational costs, it also reduces water overlap in the initial spans, which may decrease distribution uniformity. It is important to note that the financial aspects associated with emitter spacing and nozzle selection methods were beyond the scope of this study. Therefore, a cost–benefit analysis is recommended for future research.

Regarding the two selection methods, MQ_ap_ and MCd, both are subject to inherent uncertainties that warrant further investigation. For instance, as observed in the Q_reference_ and Q_measured_ analysis, actual emitter discharge may deviate from nominal values due to manufacturing tolerances or field conditions, potentially affecting the accuracy of the MQ_ap_ method. Similarly, the individual and mean discharge coefficients used in the MCd method may also vary under real-world conditions. However, despite their distinct approaches, both methodologies yielded notably similar results, suggesting that either may serve as a reliable tool for nozzle selection in practical applications and simulations. Based on these findings, MQ_ap_ stands out due to its greater theoretical simplicity and procedural ease.

The data underlying the figures and tables presented in this section (“Selected nozzles”) and the preceding one (“Measured and reference flow rates”) are provided in the supporting files (S1 File.xlsx).

### Collected water depth

**Fig 13:**
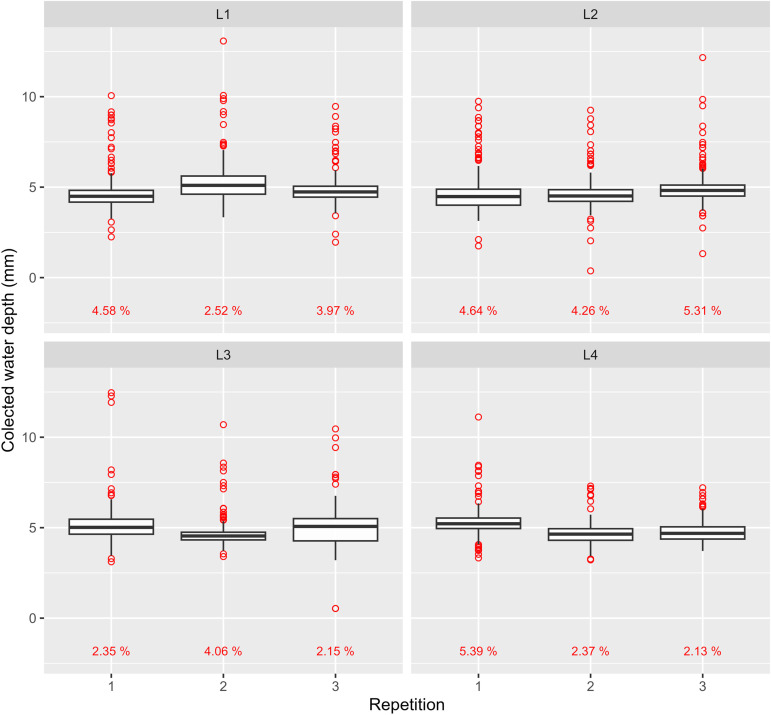
Boxplots of uniformity field test results for P180. Red dots and percentage represent outliers and their percentages regarding the number of observations, respectively.

**Fig 14:**
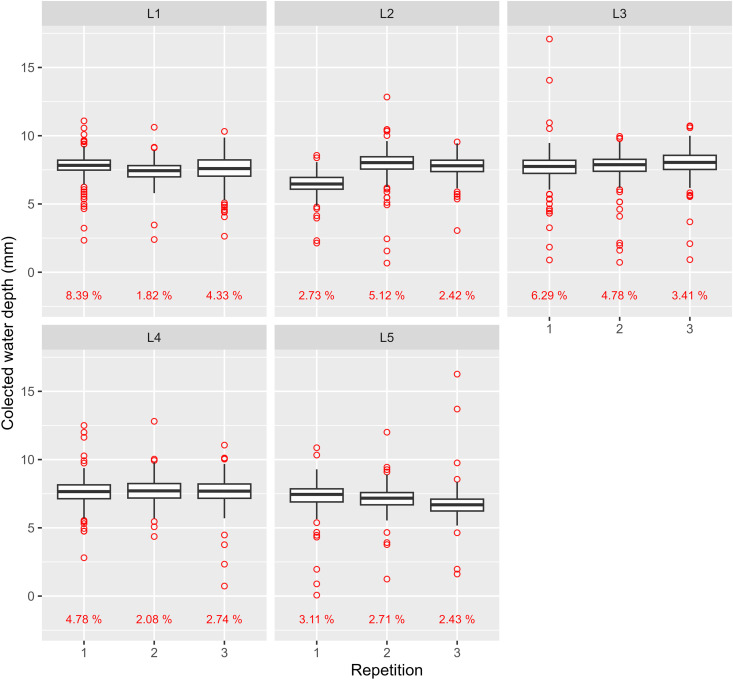
Boxplots of uniformity field test results for P360. Red dots and percentage represent outliers and their percentages regarding the number of observations, respectively.

Figs 13 and 14 present boxplots for each trial with a collector spacing of 1 m, grouped by collector line positions. The field data collected from these trials, which served as the basis for the subsequent analyses and figures, are provided in the supporting files (S2 File.xlsx).

Both figures show a small interquartile range across repetitions. However, for P360, a greater variation is observed in the first repetition at position L2 and the last repetition at L5. This variation may be attributed to undetected pressure fluctuations during the tests, in addition to climatic interferences. Electrical supply instabilities, which are common in rural areas, can cause such fluctuations, often disrupting irrigation system operation [13].

During the evaluation of L3 for P180, third repetition, rainfall occurred midway through the collector line readings. Since the test could not be repeated, the average rainfall depth recorded by a nearby pluviometer was subtracted from the remaining collectors. Despite this correction, the rain’s impact remains evident in the larger interquartile range observed in Fig 13.

The concentration of red dots above or below the whiskers may suggest a systematic pattern. For P180, elevated outliers are consistently present across all repetitions, whereas P360 exhibits a more evenly distributed outlier pattern.

With a 1-meter collector spacing, collected water volumes were expected to be more sensitive to field variations. A common observation was water dripping from emitters due to wetting by neighboring emitters. Collectors positioned directly beneath the emitter trajectory captured higher water volumes, likely contributing to more upper-limit outliers. This effect is more pronounced at larger pivot radii due to a higher probability of collectors aligning directly under emitters, and longer exposure to dripping from inner spans. Given the significantly larger radius of P180, this effect likely caused the higher concentration of elevated outliers compared to P360.

Regarding lower-limit outliers, wind interference is believed to be the primary factor, since collectors closer to the pivot point are expected to be more susceptible to wind due to the ESP_field_ configuration. It is well known that wind speed and direction can influence both distribution uniformity and drift losses; however, these effects are complex and context-dependent, as they may vary substantially across time and space. As reported by [28], wind can either improve or worsen results depending on its interaction with the lateral line (e.g., acute or obtuse angle, inward or outward flow, and direction relative to pivot movement). In the present study, since such information was not recorded during the tests, its influence on uniformity and applied water depths cannot be thoroughly examined — an acknowledged limitation also noted by [13]. Nevertheless, the experimental setup adopted in this work — based on trials conducted in two center pivots and a detailed spatial sampling design with closely spaced and repeatedly measured collector positions — was structured in such a way that environmental variability, including potential effects of wind speed and direction, could be addressed, thereby reducing its influence on treatment comparisons.

### Wind drift and evaporation losses

Table 7 presents the averaged L_W_ and DE_L_ for each analyzed position. The estimates were based on the 1-meter collector spacing dataset and projected water depths for a regulation of 50% (Table 4).

**Table 7:** Average weighted water depths (L_W_) and evaporation and drift losses (DE_L_) by collector line position.

Position		L_W_ (mm)	DE_L_ (%)
**P360**	**L1**	7.52	14.35
	**L2**	7.43	15.39
	**L3**	7.87	10.36
	**L4**	7.80	11.22
	**L5**	7.05	19.74
**P180**	**L1**	4.83	10.63
	**L2**	4.43	18.04
	**L3**	4.84	10.58
	**L4**	4.61	14.73

DE_L_ ranged from 10.36% to 19.74% for P360, averaging 14.21%, and from 10.63% to 18.04% for P180, with an average of 13.49%. Both pivots exhibited similar DE_L_ behavior, with closely matching extremes and averages, suggesting a convergence in overall DE_L_ patterns despite slightly higher values for P360. This similarity is likely due to shared factors such as daytime activation and the location of pivots on the same farm, exposing both systems to similar climatic conditions.

DE_L_ values in Table 7 appear to correlate with trends observed in Figs 13 and 14. For P180, position L2, which recorded the highest DE_L_, also had the highest outlier occurrence (Fig 13), averaging 4.74%. Additionally, higher DE_L_ values resulted in lower L_W_ values at positions L2 and L4. A similar pattern was observed for P360, where lower L_W_ values were associated with higher DE_L_, particularly at L5. However, despite L5 recording the highest DE_L_, its average outlier percentage (2.75%) was the lowest amongst all positions.

[3] evaluated eight standard pivots across different regions in Saudi Arabia for CUC_HH_ and DE_L_, reporting CUC_HH_ values between 74% and 90% while measured and predicted DE_L_ ranged from 7.5% to 15%. Similarly, [29] analyzed center pivots in Albacete, Spain, finding mean DE_L_ values of 12.5% for rotating emitters at a height of 2.5 m and 8.2% for emitters at 1 m during daytime evaluations. [30] observed significant evaporation losses of approximately 16%, attributed to high temperatures (~30°C) and low relative humidity (~40%) during daytime tests, with wind speeds below 3 m s ⁻ ¹. In this study, evaluations were conducted under similar conditions, with mean temperature, relative humidity, and wind speed of 32.96 ± 3.6°C, 39.18 ± 11.52%, and 2.32 ± 0.68 m s ⁻ ¹, respectively, aligning with [30]. However, it should be recalled that these environmental parameters were recorded only at the beginning of each test, which represents a limitation of the study.

Given the substantial DE_L_ variation in both pivots, individual tests, regardless of installation position, may differ significantly from the area’s average. Additionally, as discussed over the differences in projected and simulated water depths shown in Tables 4 and 5, DE_L_ values may be subject to some variation.

### Distribution uniformity and water depth assessment

Table 8 presents a summary of the analysis of variance (ANOVA) for both pivots.

**Table 8:** Summary of ANOVA for individual collector lines, analyzing position and spacing for average water depth (L_M_), weighted water depth (L_W_), and modified Christiansen’s uniformity coefficient (CUC_HH_).

	P180				P360			
VF^(1)^	DF^(2)^	F-statistic			DF	F-statistic		
		L_M_	L_W_	CUC_HH_		L_M_	L_W_	CUC_HH_
**Block**	2	1.86 ^NS^	2.04 ^NS^	7.33**	2	6.14**	9.12**	8.26**
**LP** ^**(3)**^	3	2.89*	6.70**	0.60 ^NS^	4	18.32**	30.72**	8.35**
**SP** ^**(4)**^	11	0.03 ^NS^	0.04 ^NS^	0.64 ^NS^	11	0.04 ^NS^	0.16 ^NS^	0.52 ^NS^
**LP x SP** ^**(5)**^	33	0.04 ^NS^	0.08 ^NS^	0.33 ^NS^	44	0.06 ^NS^	0.18 ^NS^	0.30 ^NS^
**Residual**	94				118			
**VC** ^**(6)**^ **(%)**		5.84	7.65	2.16		5.46	5.48	2.40

(1)VF: Variation factor; ^(2)^ DF: Degrees of freedom; ^(3)^ LP: Collector line positions; ^(4)^ SP: Spacing between collectors; ^(5)^ LP x SP: Combined effect of collector position and spacing; ^(6)^ VC: Variation coefficient. * significant at 5%; ** significant at 1%; ^NS^ Non-significant.

For both pivots, collector spacing (SP) from 1 to 12 meters showed no significant differences in mean values. Additionally, the interaction between collector line position (LP) and spacing (LP × SP) was not significant for any response variable. However, LP had a significant effect on L_M_ and L_W_ for P180, while for P360, differences were also found in CUC_HH_.

Table 9 presents the average values of L_M_, L_W_, and CUC_HH_, along with Tukey’s mean comparison test results for the variables that showed significance in the previous analysis (Table 8).

**Table 9:** Averages of collector line positions for mean water depth (L_M_), weighted water depth (L_W_), and modified Christiansen’s uniformity coefficient (CUC_HH_).

P180				P360			
Position	L_M_ (mm)	L_W_ (mm)	CUC_HH_ (%)	Position	L_M_ (mm)	L_W_ (mm)	CUC_HH_ (%)
L1	4.86 a	4.82 a	90.36	L1	7.56 ab	7.53 bc	93.33 a
L2	4.70 a	4.50 c	89.92	L2	7.35 b	7.40 c	92.20 ab
L3	4.87 a	4.77 ab	89.87	L3	7.81 a	7.89 a	92.10 ab
L4	4.86 a	4.56 bc	90.30	L4	7.62 a	7.79 ab	91.49 bc
				L5	7.05 c	6.92 d	90.43 c

Means followed by at least one common letter within columns do not differ significantly from each other at a 5% significance level by Tukey’s test.

### Individual analysis of collector lines for P180

Although ANOVA indicated differences in L_M_ (Table 8), Tukey’s test did not detect significant differences between values. However, for L_W_, position L1, which experiences more pronounced elevation variations, had significantly higher values than L2 and L4 but was similar to L3. Additionally, L_W_ values for L3 and L4 were statistically equal, which is expected given their proximity. Notably, the positions with the lowest L_W_ averages (L2 and L4) also exhibited the highest DE_L_ values, as shown in Table 7.

Since L_M_ remained equal across positions, the weighting factor combined with DE_L_ likely contributed to the observed differences in L_W_, with lower depths occurring toward the pivot’s end, such as at L2. Conversely, L1, which undergoes greater elevation changes than L2, showed increasing depths along the radius. The end of L1 is approximately 8 m lower than the end of L2 (Fig 4A). This elevation difference likely increased pressure along L1, resulting in greater water depths near its end and a higher L_W_, though without significantly affecting CUC_HH_. This observation aligns with the simulated total water depth based on Q_measured_ (Fig 9A), where higher flow rates were recorded beyond 300 m from the pivot center at L1.

These findings highlight the importance of pressure-regulating valves in P180. Despite operating on highly irregular terrain and being due for replacement, the valves effectively controlled pressure variations, ensuring that CUC_HH_ remained consistent across the evaluated positions, even though L_W_ varied.

### Individual analysis of collector lines for P360

For position L5, L_M_, L_W_, and CUC_HH_ had lower averages compared to other positions, except for CUC_HH_ in L4. Given L5’s proximity to L4, greater similarity in L_M_ and L_W_ was expected. However, Fig 14 shows consistently lower water depths for all L5 repetitions compared to L4, contributing to a lower mean.

During field tests, the lateral line moved counterclockwise, initially irrigating L5. This suggests that wind drift primarily contributed to higher L_M_ and L_W_ at L4, at the expense of lower values at L5. Supporting this, as shown in Table 7, DE_L_ was considerably higher for L5 despite its proximity to L4. However, since wind data were only collected at the beginning of the test, a broader analysis of wind conditions during the evaluations was not feasible.

For L2, despite being the only position with significant elevation differences (Fig 4B), where its end is approximately 5 meters below the pivot point, CUC_HH_ values were similar to those of other positions, except for L5. Additionally, L_M_ and L_W_ were statistically equivalent to L1, a diametrically opposite leveled line, suggesting that the slope at L2 might have not significantly impacted the results.

Overall, greater variation in L_M_, L_W_, and CUC_HH_ was observed based on LP for P360 compared to P180. This was unexpected given the significantly more irregular terrain of P180. This discrepancy may be due to unrecorded climatic variations at the P360 site during testing, particularly wind, as P360 is in a much flatter area. Consistently, DE_L_ (Table 7) had a wider range and a higher average for P360 than P180. Additionally, the significance of the blocking factor supports this unexpected result, suggesting that external and uncontrolled factors — such as varying climatic conditions between repetitions — likely influenced L_M_, L_W_, and CUC_HH_ measurements for P360. In contrast, for P180, the blocking factor was significant only for CUC_HH_.

### Grouped analysis of collector lines

Table 10 displays the summary of ANOVA results for the methods MA_4 lines_, MA_3 lines_, adapted MA_3 lines*_, MA_ABNT_, and MA_single_ used to assess L_M_, L_W_, and CUC_HH_. Since SP was not significant (Table 8), only field data from tests with collectors spaced 1 meter apart were used to ensure an analysis focused on the most representative and detailed dataset.

**Table 10:** Summary of ANOVA for grouped collector lines for average water depth (L_M_), weighted water depth (L_W_), and modified Christiansen’s uniformity coefficient (CUC_HH_).

	P180				P360			
VF^(1)^	DF^(2)^	F-statistic			DF	F-statistic		
		L_M_	L_W_	CUC_HH_		L_M_	L_W_	CUC_HH_
**Block**	2	2.79 ^NS^	2.41 ^NS^	3.89 ^NS^	2	1.70 ^NS^	0.31 ^NS^	3.06 ^NS^
**Methods**	2	0.16 ^NS^	0.43 ^NS^	0.06 ^NS^	3	0.94 ^NS^	1.24 ^NS^	4.14 ^NS^
**Residual**	4				6			
**VC** ^**(3)**^ **(%)**		3.68	4.04	1.22		3.01	3.16	0.40

(1)VF: Variation factor; ^(2)^ DF: Degrees of freedom; ^(3)^ VC: Variation coefficient; * significant at 5%; ** significant at 1%; ^NS^ Non-significant.

The ‘Methods’ factor, which includes various collector line positions in the field, showed no significant differences among mean values for the three analyzed variables. Table 11 presents L_M_, L_W_, and CUC_HH_ values for each method used. While Table 9 indicated differences in L_M_, L_W_, and CUC_HH_ for P360 and L_W_ for P180 across individual collector line positions, these differences were reduced when grouped by ‘Methods’.

**Table 11:** Average values of evaluation methods for average water depth (L_M_), weighted water depth (L_W_), and modified Christiansen’s uniformity coefficient (CUC_HH_).

P180				P360			
Methods	L_M_ (mm)	L_W_ (mm)	CUC_HH_ (%)	Methods	L_M_ (mm)	L_W_ (mm)	CUC_HH_ (%)
MA_3 lines_	4.81	4.70	90.59	MA_4 lines_	7.60	7.65	92.51
MA_ABNT_	4.88	4.73	90.58	MA_3 lines_*	7.52	7.61	92.31
MA_single_	4.89	4.84	90.31	MA_ABNT_	7.37	7.42	91.57
				MA_single_	7.66	7.80	91.80

MA_single_: a single leveled line; MA_ABNT_: two lines angled at 3° relative to each other; MA_3 lines_: three lines, with two subjected to both uphill and downhill terrain, and one on leveled ground; MA_3 lines_*: modified version applied to P360 using only two lines due to the absence of an uphill area; MA_4 lines_: four lines arranged perpendicularly.

As highlighted by [13], while radially arranged collector lines are more responsive to flow rate variations along the lateral line, they may not accurately represent conditions across most of the irrigated area due to their limited, one-directional sampling. However, except for MA_single_, all evaluation methods reflect the average L_M_, L_W_, and CUC_HH_ values from multiple collector lines. Increasing the number of radially installed collector lines at different positions enhances representativeness, providing more accurate estimates of water depths and CUC_HH_ for the entire irrigated area.

In some cases, uniformity values for combined collector line methods (Table 11) were slightly higher than those for isolated lines (Table 9), a pattern previously noted by [2]. This occurs because combining different collector lines helps dilute individual variations that would otherwise reduce uniformity values.

Given the material and labor required for uniformity assessments, MA_single_ is the most efficient method for general evaluations of P180 and P360. However, as previously discussed for P180, position L1, assessing locations with the greatest elevation variations along the radius is recommended to ensure a more precise evaluation of water depth.

The P-values corresponding to the statistical analyses presented in Tables 8 and 10 are provided in the supporting files (S3 File.xlsx).

### Additional discussion

The CUC_HH_ values in this study averaged above 90%, classifying them as excellent (Table 3). [31] analyzed 21 pivots across various regions in Minas Gerais, Brazil, and found CUC_HH_ values mostly above 80%. Similarly, [32] reviewed studies from Spain and the USA, where CUC_HH_ ranged from 49% to 93%. [33] evaluated 16 pivots in Goiás and Distrito Federal, Brazil, with values between 60% and 93%, highlighting that, in some cases, oversized nozzles and damaged pressure regulators contributed to excessive water depths.

[12] assessed water depth, distribution uniformity, and energy consumption for collector lines positioned uphill, downhill, and on level terrain in a pivot equipped with a variable frequency drive. They concluded that position influenced results, though CUC_HH_ remained above 85% likely due to properly functioning pressure-regulating valves and the water overlap provided by rotating emitters. In a similar study analyzing water depth and uniformity across downhill, level, and uphill positions with a 24.37-meter elevation difference, [30] found no significant variation between positions. This stability was also attributed to the proper functioning of the pressure-regulating valves. In comparison, P180 and P360 performed exceptionally well, approaching the highest reported uniformity values. However, water depths varied, aligning with findings from previous studies.

[34] assessed CUC_HH_ using complete data sets, as well as subsets containing half and one-third of the data in order to simulate different collector spacings, and found that the spacing appears to have no considerable effect on CUC_HH_. They also noted that larger spacings may assess overall system performance, whereas shorter ones help identify localized issues. In line with those findings, the present study systematically examined a broader range of collector spacings and found no significant influence on CUC_HH_. This insensitivity is likely due to the high distribution uniformity of the two evaluated pivots, which are regularly maintained and operated by specialized personnel — a standard that should ideally be met in practice. Although the analysis encompassed leveled areas, downhill positions, and zones with mixed slope characteristics, no evaluated location was situated entirely uphill. While this does not invalidate the results, it may partially limit extrapolations to systems installed under more complex topographical conditions, where terrain-induced pressure losses could meaningfully affect water distribution uniformity, potentially making wider collector spacings less appropriate.

These findings suggest practical opportunities to simplify field evaluations by supporting wider collector spacings without compromising the reliability of the results. Future studies could expand upon this by assessing central pivots equipped with different emitters, since this work evaluated a single emitter model (I-Wobler). They could also investigate less optimized systems, including those with greater topographic variation, suboptimal pressure conditions, or issues related to water quality such as emitter clogging, in order to refine spacing recommendations across a broader range of irrigation contexts.

## Conclusions

The conclusions are specific to the evaluated conditions, and results may vary with different equipment or configurations. Therefore, under the analyzed conditions, it can be concluded that:

- Uniformity evaluation methods, which combine various collector line positions, showed no significant differences in mean values.
- Spacing (1 to 12 meters) and its interaction with the collector line position did not significantly influence the results.
- L_W_, L_M_, and CUC_HH_ values varied significantly across collector line positions for P360, whereas for P180, differences were observed only for L_W_.
- Nozzle packages obtained through MQ_ap_ and MCd methods for both ESP_constant_ and ESP_field_ resulted in higher theoretical CUC_HH_ values than those simulated based on observed nozzle listings in the field for both pivots.
- Total water depths were higher, while simulated CUC_HH_ values using Q_measured_ were lower compared to those simulated with Q_reference_.
- Average CUC_HH_ values for both pivots were classified as excellent, while evaporation and drift losses were comparable to the highest values reported in the literature.

## Supporting information

S1 File Flow rate measurements and nozzle simulations. (XLSX)

S2 File Uniformity field data (1m). (XLSX)

S3 File ANOVA p-values. (XLSX)
